# Porcelain Teeth

**Published:** 1853-01

**Authors:** 


### PORCELAIN TEETH.

We have on hand specimens of teeth from the manufactory of Mr. John Klein, Philadelphia. The body appears to have been carefully prepared, possessing strength and transparency, and the platina pins of a good size; so far as we have tested them they stand the heat of the blowpipe very well. The grinding surface of the molars and bicuspids are carved with more than ordinary care, and we think more naturally formed than the labial face of the teeth. The coloring of the teeth is very perfect, and the gum color is very natural. We refer the Profession to his card in our advertising sheet, and we have no doubt but that Mr. Klein's enterprise will soon be duly rewarded with an extensive patronage.

